# Application of Biosheets as Right Ventricular Outflow Tract Repair Materials in a Rat Model

**DOI:** 10.3389/fvets.2022.837319

**Published:** 2022-04-08

**Authors:** Takeshi Mizuno, Ryosuke Iwai, Takeshi Moriwaki, Yasuhide Nakayama

**Affiliations:** ^1^Veterinary Medical Center, Graduate School of Agricultural and Life Sciences, The University of Tokyo, Tokyo, Japan; ^2^Research Institute of Technology, Okayama University of Science, Okayama, Japan; ^3^Department of Mechanical Science and Engineering, Faculty of Science and Technology, Hirosaki University, Aomori, Japan; ^4^Osaka Laboratory, Biotube Co., Ltd., Osaka, Japan

**Keywords:** in-body tissue architecture, congenital heart defect, rat model, tissue engineering, autologous tissue membrane

## Abstract

**Purposes:**

We report the experimental use of completely autologous biomaterials (Biosheets) made by “in-body tissue architecture” that could resolve problems in artificial materials and autologous pericardium. Here, Biosheets were implanted into full-thickness right ventricular outflow tract defects in a rat model. Their feasibility as a reparative material for cardiac defects was evaluated.

**Methods:**

As the evaluation of mechanical properties of the biosheets, the elastic moduli of the biosheets and RVOT-free walls of rats were examined using a tensile tester. Biosheets and expanded polytetrafluoroethylene sheet were used to repair transmural defects surgically created in the right ventricular outflow tracts of adult rat hearts (*n* = 9, each patch group). At 4 and 12 weeks after the operation, the hearts were resected and histologically examined.

**Results:**

The strength and elastic moduli of the biosheets were 421.3 ± 140.7 g and 2919 ± 728.9 kPa, respectively, which were significantly higher than those of the native RVOT-free walls (93.5 ± 26.2 g and 778.6 ± 137.7 kPa, respectively; *P* < 0.005 and *P* < 0.001, respectively). All patches were successfully implanted into the right ventricular outflow tract-free wall of rats. Dense fibrous adhesions to the sternum on the epicardial surface were also observed in 7 of 9 rats with ePTFE grafts, whereas 2 of 9 rats with biosheets. Histologically, the vascular-constructing cells were infiltrated into Biosheets. The luminal surfaces were completely endothelialized in all groups at each time point. There was also no accumulation of inflammatory cells.

**Conclusions:**

Biosheets can be formed easily and have sufficient strength and good biocompatibility as a patch for right ventricular outflow tract repair in rats. Therefore, Biosheet may be a suitable material for reconstructive surgery of the right ventricular outflow tract.

## Introduction

Approximately 0.3% of infants require corrective surgery for congenital heart defects (CHDs) within their first year of life (1). Synthetic or animal tissue-based materials, such as expanded polytetrafluoroethylene (ePTFE), polyethylene terephthalate, or glutaraldehyde-treated xenopericardium, have been widely used as patch materials for repairing CHD. However, these materials have several drawbacks. First, they are associated with rejection, stenosis, aneurysm formation, and calcification (2–6). Moreover, because these materials are nonliving, they do not grow, and remodeling cannot occur. Although autologous pericardium shows good potential as a patch for repairing CHD, it cannot be used for multiple cardiac surgeries because of size limitations.

We have previously developed autologous prosthetic tissues using “*in-body* tissue architecture (IBTA)” technology, a novel and practical approach for regenerative medicine based on the tissue encapsulation phenomenon of foreign materials in living bodies (7). This technology involves the use of living bodies as a reactor and does not require expensive facilities or complicated manipulations. We have reported the construction of completely autologous tissues, such as tri-leaflet heart valves (i.e., biovalves) (8–24), vascular grafts (i.e., biotubes) (25–35), and membranous tissues (i.e., biosheets) (36–41), using this technology. Tissue prostheses prepared using the IBTA technology were reconstructed based on the recipient tissues after implantation. For example, biotubes implanted into the native aorta in rat and rabbit models were reconstructed into vascular tissues and were completely endothelialized, with multiple layers of smooth muscle cells and dense collagen fibers exhibiting a regular, circumferential orientation (25, 30, 31). The random orientation of collagen fibrils in the original biosheets, which were implanted in the rabbit corneal stroma, tended to be homogeneous, similar to that of the native stroma (41). Therefore, tissue prostheses prepared using the IBTA technology may represents alternative biomaterials with high potential to overcome the abovementioned problems encountered with synthetic or animal tissue-based materials.

Tissue prostheses can be fabricated in various shapes and sizes to suit the needs of individual recipients. In contrast to autologous pericardium, biosheets can be used as patches for multiple reconstructive cardiac surgeries. Additionally, biotubes and biovalves have sufficient strength to withstand arterial pressure over 1,000 mmHg and have been successfully implanted as cardiovascular replacements without rupture. However, the capacity for the application of biosheets in the repair of CHDs is unknown.

This study aimed to assess the potential of biosheets as a reparative material for cardiac defects. We partially resected the right ventricular outflow tract (RVOT) of adult rats and repaired the defect using either biosheets or ePTFE sheets (control). The patches were examined histologically over a 12-week period.

## Materials and Methods

### Animal Studies

Wistar rats (*n* = 18, approximately 300–350 g) were used in this study performed at the National Cerebral and Cardiovascular Center Research Institute. All animals received care according to the Principles of Laboratory Animal Care. All animal studies were performed in accordance with the Guide for the Care and Use of Laboratory Animals published by the US National Institutes of Health (NIH Publication No. 85-23, revised 1996) under a protocol approved by the National Cerebral and Cardiovascular Center Research Institute Committee (No. 13034).

### Preparation of Biosheets

**Figure 1:**
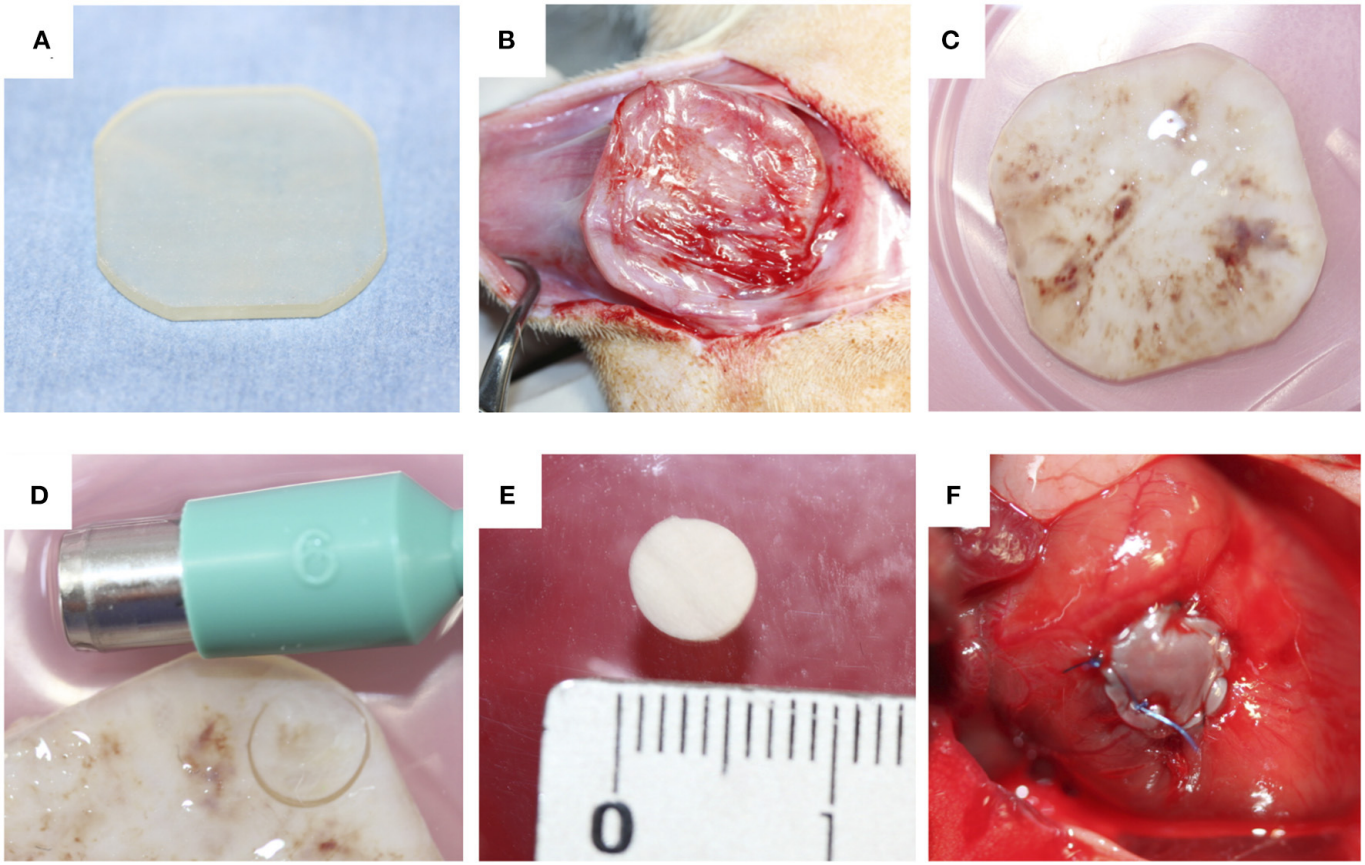
**(A)** Acrylic plates (25 mm × 25 mm × 2 mm) were used as a mold. **(B,C)** The molds were completely encapsulated with connective tissues. **(D)** Pieces of the biosheets were obtained by biopsy punch **(E)**. The obtained biosheet **(F)**.

Acryl plates (25 mm × 25 mm × 2 mm) were prepared using a 3D printer (CONNEX 260, Objet, Rehovot, Israel; Figure 1A). Anesthesia was induced and maintained by isoflurane inhalation, and a 3-cm incision was made in the shaved dorsal skin of anesthetized rats. A plate was placed in the dorsal subcutaneous pouch. After 4 weeks, the implants encapsulated with connective tissues were harvested under isoflurane anesthesia (Figure 1B). Biosheets were obtained as connective tissue membranes after removing the plates (Figure 1C). Circular biosheets with a diameter of 6 mm for implantation were obtained by cutting them with a biopsy punch (Figures 1D,E). The remaining tissues were used for histological evaluations and mechanical tests.

### Mechanical Properties

The elastic moduli of the biosheets and RVOT-free walls of rats were examined using a tensile tester (P&M, Fukushima, Japan) by using the same method as previously reported (35). Tissue specimens (10 mm × 10 mm) were tested under humid conditions. The load was recorded until the samples ruptured, with a tissue extension rate of 0.05 mm/s. The elastic modulus values were obtained from the maximum slope of the deformation-force relationship.

### RVOT-Free Wall Resection and Replacement

The surgical procedure was performed according to previously reported methods (42). Briefly, rats were anesthetized with isoflurane. Intubation was performed with ventilation at 60 cycles/min under room air supplemented with oxygen (2 L/min) and 1.5%−2.5% isoflurane. The heart was exposed through median sternotomy. A purse-string suture was then placed in the RVOT-free wall with 7-0 polypropylene to form a perimeter >6 mm in diameter. Both suture ends were passed through a 22-gauge plastic venous cannula, which was used as a tourniquet, and the tourniquet was tightened. The RVOT wall inside the purse-string suture was distended and resected to create a defect <6 mm in diameter. The tourniquet was briefly loosened to assess pulsatile bleeding, confirming the formation of a transmural defect. The circular biosheet or ePTFE patch (diameter, 6 mm; thickness, 0.4 mm) was sutured along the margin of the purse-string suture with 7-0 polypropylene to cover the defect (Figure 1F). The biosheet was sutured with the smooth side facing the RVOT lumen. The tourniquet was then released, and the purse-string suture was removed. The chest incision was closed in layers with simple continuous sutures using 3-0 nylon. After the surgery, the rats were monitored in a warm environment until they had completely recovered from anesthesia. The rats were then returned to their cages. Anticoagulation therapy was not administered.

### Histological and Immunohistochemical Analysis

At each scheduled explant period (4 and 12 weeks after surgery, *n* = 3 and *n* = 6 in each group, respectively), animals were administered 300 units of heparin intravenously and were then euthanized by intravenous injection of an overdose of pentobarbital (100 mg/kg). The heart was exposed by repeated median sternotomy and fixed overnight with 4% paraformaldehyde. The RVOT-free wall around the patch implantation site was resected and used for histological evaluation. The overall appearance of the endocardial surface was examined. The sections were stained with hematoxylin and eosin or Masson's trichrome or stained immunohistochemically. Anti-CD31 monoclonal antibodies (1:100; Abcam, Cambridge, UK) were used to identify the endothelial cells. Sections were incubated with primary antibodies overnight at 4°C in 1% bovine serum albumin followed by washing with phosphate-buffered saline (PBS). AlexaFluor secondary antibodies (1:1,000; A-11012, Life Technologies, Carlsbad, CA, USA) were used for 2 h at room temperature, followed by washing with PBS. Nuclei were counterstained with DAPI (4′,6-diamidino-2-phenylindole).

### Statistical Analysis

Statistical analysis was performed using the Mann–Whitney U test to compare the strength and elastic moduli between those of the native RVOT-free walls and biosheets. Statistical analyses were performed using a commercially available software (SPSS 23.0, IBM Inc., Armonk, NY, USA). Statistical significance was set at *P* < 0.05.

## Results

### Preparation of Biosheets

After 4 weeks within the dorsal subcutaneous pouches, acrylate plates were completely covered with a thin connective tissue membrane, which was considered the biosheet (Figure 1C). Although the outer surface of the biosheet was delicately connected with subcutaneous tissues through an irregularly shaped surface, the internal surface of the biosheet exhibited a smooth, flat surface in contact with the plate (Figure 1E).

**Figure 2:**
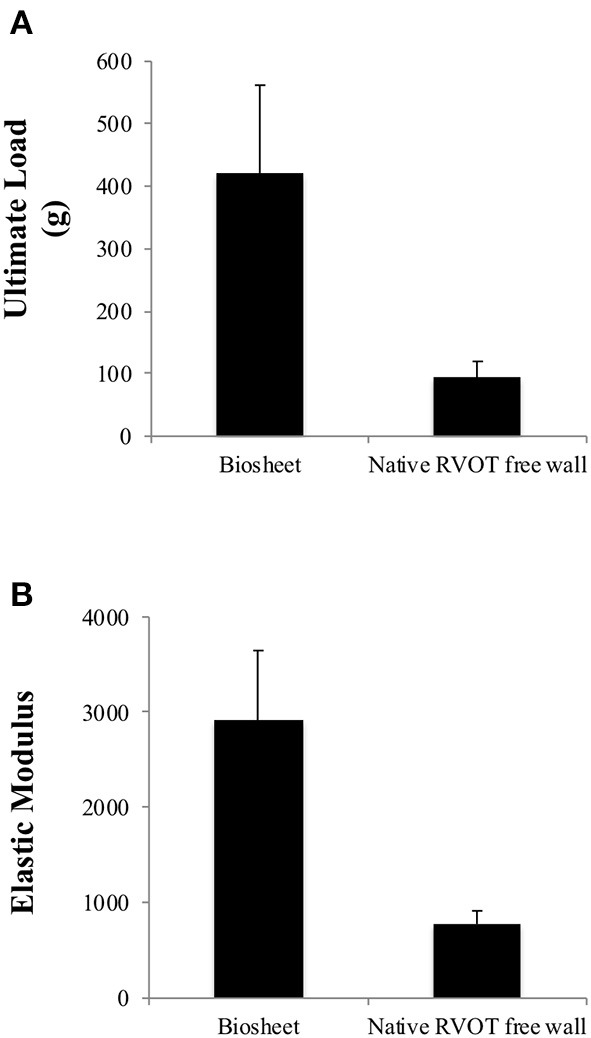
Comparison of the ultimate load **(A)** and elastic moduli **(B)** of the biosheets and native RVOT-free wall. The strength and elastic moduli of the biosheets were significantly higher than those of the native RVOT-free walls.

The strength and elastic moduli of the biosheets were 421.3 ± 140.7 g and 2,919 ± 728.9 kPa, respectively, which were significantly higher than those of the native RVOT-free walls (93.5 ± 26.2 g and 778.6 ± 137.7 kPa, respectively; *P* < 0.005 and *P* < 0.001, respectively; Figures 2A,B).

### Resection and Replacement of the RVOT-Free Wall

**Figure 3:**
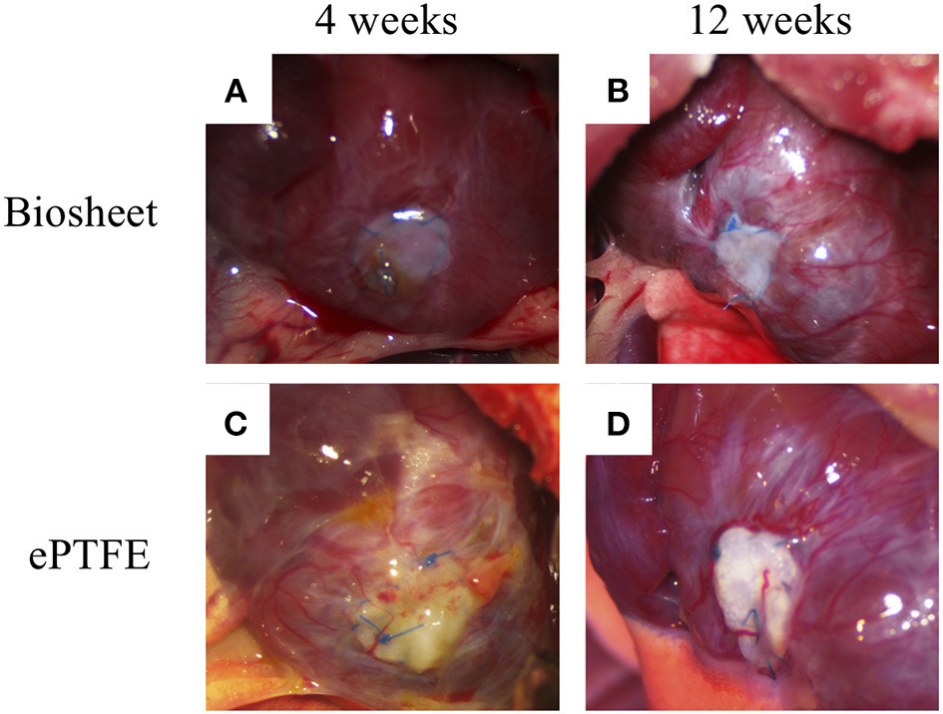
Representative images of biosheets **(A,B)** and ePTFE **(C,D)** at cardiac explantation. Dense fibrous adhesions to the sternum on the epicardial surface were also observed in 7 of 9 rats with ePTFE grafts, whereas 2 of 9 rats with biosheets.

Biosheets were successfully implanted into the RVOT-free walls of rats. None of the rats died from the procedure (Figure 1F), and all rats survived until the scheduled euthanasia. Furthermore, no animals showed signs of infection after the implantation. Dense fibrous adhesions to the sternum on the epicardial surface were also observed in 7 of 9 rats with ePTFE grafts, whereas 2 of 9 rats with biosheets (Figures 3A–D). The two types of patches were surrounded by layered fibrous tissue, and no thrombi were observed on the endocardial surface of the patches in either group at any time point. The thickness of the biosheet measured via microscope on a sample after staining did not decrease at 4 and 12 weeks after transplantation (284 ± 56 μm and 297 ± 37 μm, respectively), and no aneurysm formation or rupture was observed.

**Figure 4:**
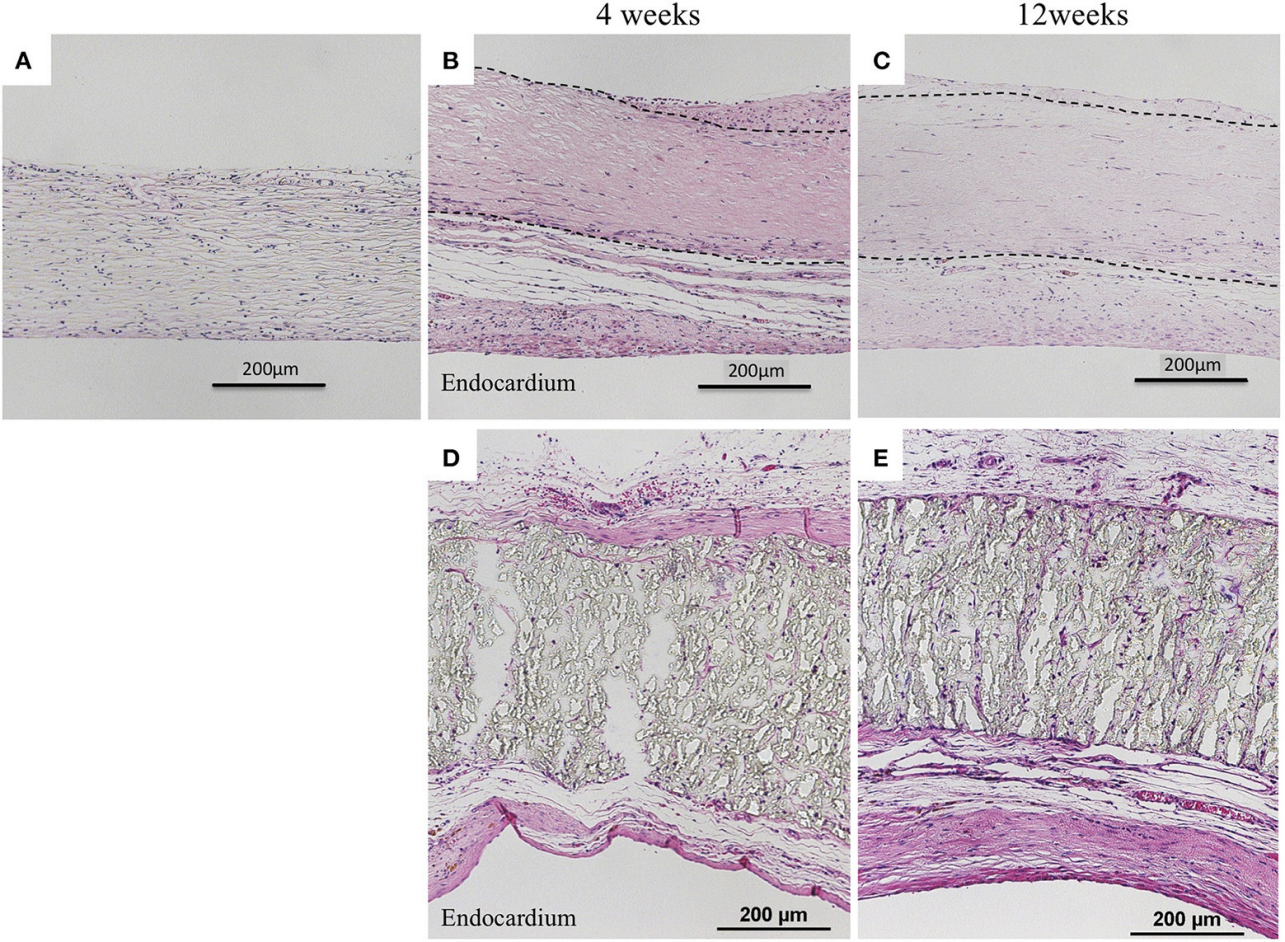
Results of histological examination (H&E staining). **(A)** Image before implantation of biosheet. Images at 4 and 12 weeks after implantation of biosheets **(B,C)** and ePTFE **(D,E)** patches. Biosheets exhibited cellular and capillary ingrowth.

**Figure 5:**
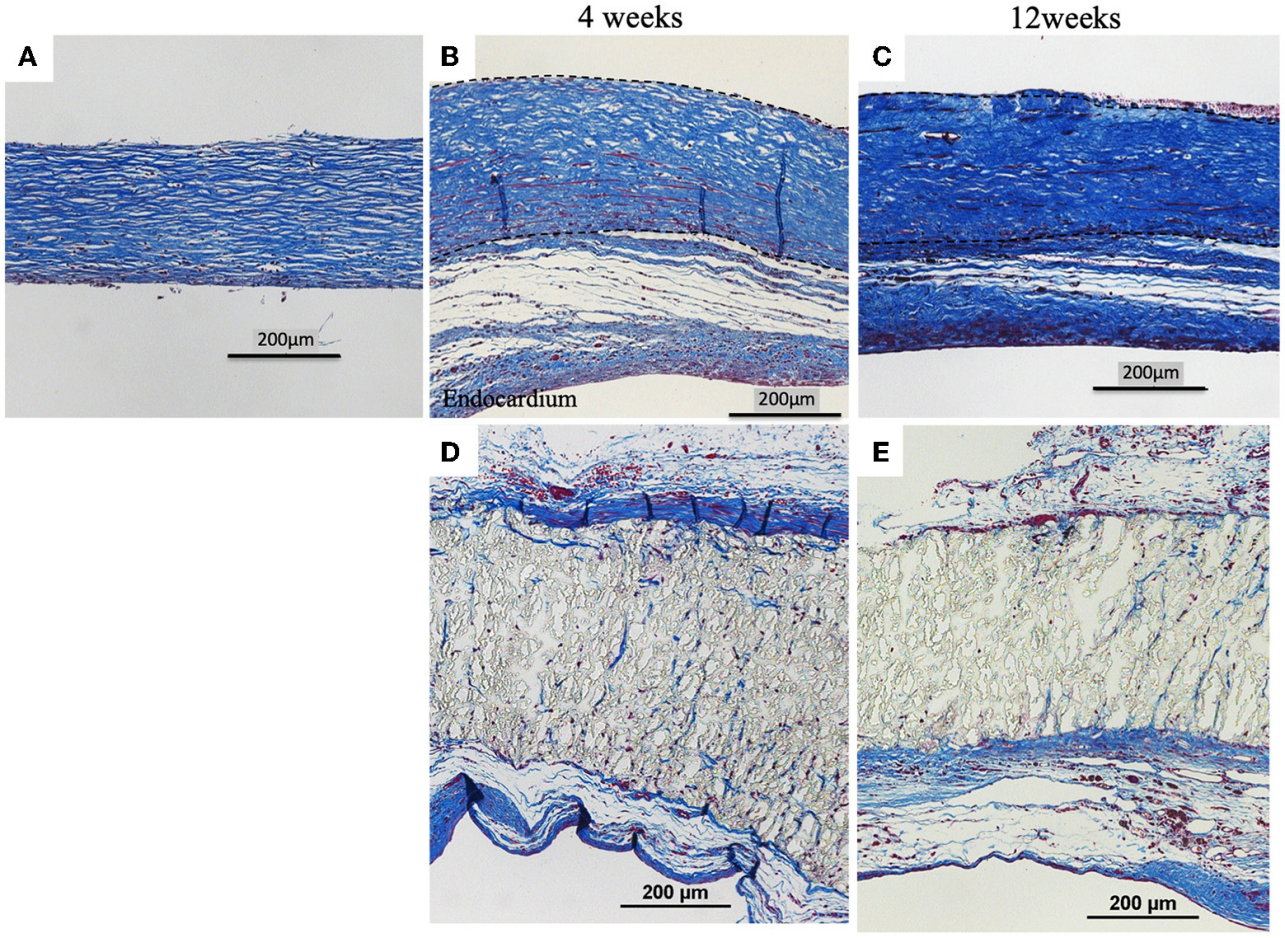
Results of histological examination (Masson's Trichrome Stain). **(A)** Image before implantation of biosheet. Images at 4 and 12 weeks after implantation of biosheets **(B,C)** and ePTFE **(D,E)** patches. Biosheets had higher collagen density at explantation than at pre-implantation.

Biosheets exhibited cellular and capillary ingrowth (Figures 4B,C, 5B,C), while there was no cellular and capillary ingrowth into the ePTFE patches (Figures 4D,E, 5D,E).

**Figure 6:**
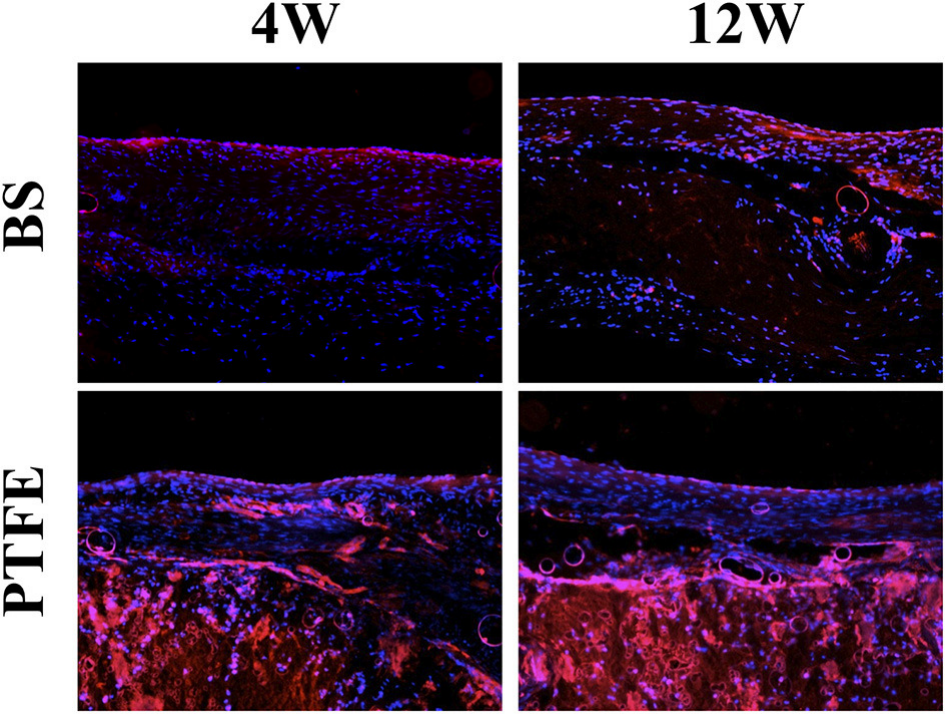
Results of histological examination (stained for vWF). Both patches had complete endothelialization on the endocardial surface in the RVOT-free wall at each time point.

Both patches had complete endothelialization on the endocardial surface in the RVOT-free wall at each time point (Figure 6).

## Discussion

Although various materials have been used as reconstructive materials for congenital cardiac diseases, each type of material has challenges, and an optimal material for the repair of CHDs has not yet been developed. Autologous pericardium is considered one of the more suitable materials for reconstructive surgeries for CHDs. However, multiple surgeries are sometimes needed to repair CHDs (43, 44), and autologous pericardium cannot be used for multiple cardiac surgeries because of size limitations. Artificial materials, such as Dacron or ePTFE patches, are also used as reparative materials for congenital cardiac diseases. These artificial materials show good results in short-term studies and are not associated with any size limitations (45, 46). Re-operation is sometimes necessary to remove stenosis because nondegradable artificial materials do not have growth potential (45). Since biosheets are generated using autologous tissues, they are not expected to be associated with this kind of problem. This study demonstrates the short-term application of biosheets as a small RVOT patch in a rat model.

Our previous studies showed that biovalves and biotubes, which are tissue prostheses prepared using IBTA, both have higher elastic moduli and stronger maximum tensile strength than native aortic valves and native arteries (8–35). Additionally, these tissue prostheses showed sufficient strength after implantation *in vivo* (8–35). In the present study, biosheets showed higher elastic moduli and stronger maximum tensile strength *in vitro* and were easily implanted into RVOT-free walls. Moreover, the biosheets did not cause varicose deformity or rupture after implantation. These results revealed that biosheets had sufficient strength to be used as a reparative material for RVOT.

Multiple surgical procedures are sometimes required to repair CHD (43, 44). As described above, autologous pericardium cannot be used for multiple surgeries because of size limitations. In contrast, biosheets can be made as many times as needed and can be formed into various shapes and sizes. In this study, all acrylic plates (25 mm × 25 mm × 2 mm) were completely encapsulated with connective tissues in rats weighing approximately 350 g. This size encapsulated the entire rat heart. A previous study confirmed that various sizes and forms of tissue prostheses prepared using IBTA could be formed in rats, rabbits, dogs, goats, and humans (8–41). Thus, we expect that appropriately sized biosheets may be used as reparative materials for cardiac diseases in humans. Furthermore, because CHD can exhibit many morphological variations (47), biosheets, which may be shaped and sized accordingly, may have great advantages as a reparative material for congenital heart disease.

The ePTFE is widely used as a cardiac patch and conduit because of its good biocompatibility (45, 46, 48). In this study, 4 weeks after implantation, endocardial surfaces of both ePTFE- and biosheet-patched hearts were covered with collagenous tissue and were completely endothelialized. Host cell ingrowth was also confirmed for biosheets. Therefore, biosheets may have good biocompatibility as well as ePTFE.

In this study, the thickness of the biosheet did not decrease at 4 and 12 weeks after transplantation, and no aneurysm formation or rupture was observed, suggesting that the biosheet maintains sufficient durability as a right ventricular restoration material for at least 12 weeks. In a previous study, we confirmed a high long-term patency rate and remodeling to an artery-like structure after implantation (49). In the current study, we observed capillary vascular and cellular ingrowth into the biosheets. Therefore, biosheets are expected to maintain long-term durability as cardiac repair materials.

Similar to previous studies that investigated the replacement of the rat RVOT, one limitation of this study was its short duration (50, 51). Because of the short duration of the study, we could not assess the growth potential or cardiac function after implantation. Additionally, we were unable to evaluate the long-term dilation resistance. Therefore, further studies are required to investigate these parameters.

Another limitation of this study is that we did not reproduce in the model the hemodynamic conditions of congenital heart disease where biosheet would be used in the real world.

Biosheets can be formed easily and have sufficient strength and biocompatibility for use as a patch for the repair of RVOT in a rat model. Therefore, biosheets may be suitable materials for reconstructive surgery for RVOT in other species. Additional long-term studies in a large animal model of myocardial patching are required to demonstrate whether Biosheets will actually grow with the host animal and whether myocyte ingrowth occurs sufficiently for cardiac contraction to happen in the patched region.

## Data Availability Statement

The original contributions presented in the study are included in the article/supplementary material, further inquiries can be directed to the corresponding author/s.

## Ethics Statement

The animal study was reviewed and approved by the National Cerebral and Cardiovascular Center Research Institute Committee.

## Author Contributions

TMi conceived and designed the study, carried out the surgical procedure, performed the statistical analysis and drafted the manuscript. RI carried out the immunostaining. TMo carried out the assessment of mechanical properties. YN participated in coordination and helped to draft the manuscript. All authors read and approved the final manuscript.

## Conflict of Interest

YN was employed by Osaka Laboratory, Biotube Co., Ltd. The remaining authors declare that the research was conducted in the absence of any commercial or financial relationships that could be construed as a potential conflict of interest.

## Publisher's Note

All claims expressed in this article are solely those of the authors and do not necessarily represent those of their affiliated organizations, or those of the publisher, the editors and the reviewers. Any product that may be evaluated in this article, or claim that may be made by its manufacturer, is not guaranteed or endorsed by the publisher.
